# Improved Iron Overload with Pegcetacoplan in Eculizumab-Experienced Patients with Paroxysmal Nocturnal Hemoglobinuria †

**DOI:** 10.3390/ijms262010019

**Published:** 2025-10-15

**Authors:** Jamile Shammo, Peter Hillmen, Peter Blandino, Vijay Abilash, David J. Kuter

**Affiliations:** 1 Feinberg School of Medicine, Northwestern University, Chicago, IL 60612, USA; 2 Apellis Pharmaceuticals, Inc., Waltham, MA 02451, USA; 3 Trinity Life Sciences, Waltham, MA 02451, USA; 4 Center for Hematology, Massachusetts General Hospital, Harvard Medical School, Boston, MA 02114, USA

**Keywords:** complement inhibitor, eculizumab, ferritin, hepcidin, iron, pegcetacoplan, paroxysmal nocturnal hemoglobinuria (PNH), transferrin

## Abstract

Complement factor 5 (C5) inhibitors for paroxysmal nocturnal hemoglobinuria (PNH) may cause iron overload due to residual intravascular hemolysis (IVH) and emergent extravascular hemolysis (EVH). In PEGASUS (phase 3; NCT03500549), adults with PNH with residual anemia (hemoglobin concentration < 10.5 g/dL) after ≥3 months of eculizumab received eculizumab and pegcetacoplan for 4 weeks and were then randomized (1:1) to eculizumab or pegcetacoplan monotherapy for 16 weeks; in the following 32-week, open-label period, patients either continued pegcetacoplan or switched from eculizumab to pegcetacoplan. This post hoc analysis reports PEGASUS transfusion-related data and iron-related biomarkers to evaluate pegcetacoplan’s effects on iron regulation. Of 80 patients randomized in PEGASUS, 27 (33.8%) had baseline iron overload (serum transferrin saturation ≥ 50%). Iron overload resolved within 52 weeks of pegcetacoplan treatment in 16 of 22 patients (72.7%) with baseline and postbaseline data; 10 experienced resolution after 20 weeks. With pegcetacoplan, transfusion numbers decreased for patients with and without iron overload, hepcidin concentrations increased, and absolute reticulocyte counts (ARCs) decreased to normal range. Mean ferritin concentrations were above normal throughout the study, regardless of iron overload status. Pegcetacoplan improves iron overload-related biomarkers, including increased hepcidin concentrations and decreased ARCs, by blocking IVH and EVH and preventing anemia.

## 1. Introduction

Paroxysmal nocturnal hemoglobinuria (PNH) is a rare, acquired hematopoietic stem cell disease that makes affected red blood cells prone to hemolysis (leading to anemia that causes fatigue and often requires transfusions) and promotes thrombosis (the primary cause of mortality in untreated PNH) [1,2]. Without treatment, patients with PNH have a survival duration of only 10–20 years after diagnosis [3].

Complement inhibitors, the current standard of care for PNH, now allow patients to have improved life expectancies [3,4,5]. The first complement inhibitors to be introduced were eculizumab and subsequently ravulizumab; both are complement factor 5 (C5) inhibitors that block the terminal portion of the complement pathway to stop the intravascular hemolysis (IVH) of PNH red blood cells, increase hemoglobin concentrations, and reduce the transfusion burden [6,7,8]. However, C5 inhibitor–treated patients often remain anemic as a result of residual IVH from incomplete C5 inhibition [9], as well as the emergence of increased extravascular hemolysis (EVH) in the liver and spleen due to ongoing proximal complement pathway activity that opsonizes surviving PNH red blood cells, targeting them for destruction [10,11]. This EVH is clinically manifested as a positive direct antiglobulin test for complement factor 3 (C3) fragments, increased indirect bilirubin concentrations [12], suboptimal hemoglobin concentrations, increased absolute reticulocyte counts (ARCs), and continued transfusion needs [13].

These manifestations of residual IVH and emergent EVH can be addressed with pegcetacoplan, the C3/C3b-targeted inhibitor that acts at a central point in the complement cascade [14,15]. In the phase 3 PEGASUS trial (NCT03500549), pegcetacoplan increased hemoglobin concentrations and reduced transfusion requirements in patients with PNH and hemoglobin < 10.5 g/dL who had received eculizumab for an average of 3–4 years [14]. The recently approved factor B inhibitor iptacopan (as monotherapy) and factor D inhibitor danicopan (as add-on therapy) also improve outcomes in C5 inhibitor–experienced patients with PNH and residual anemia; both treatments inhibit the alternative pathway, one of the activation pathways of the proximal complement cascade [16,17].

An understudied yet potentially serious effect of PNH is iron metabolism dysregulation. Without treatment, the uncontrolled IVH of PNH red blood cells leads to iron deficiency. Together with increased erythropoiesis (mediated in part by increased erythropoietin, which in turn increases erythroferrone, an inhibitor of hepcidin transcription [18]), this iron deficiency suppresses the hepatic production of the hormone hepcidin, an inhibitor of the iron-export protein ferroportin 1 [19]. The reduced inhibition of ferroportin 1 increases iron absorption and iron release from macrophages to support compensatory red blood cell production [19,20]. However, despite increased iron absorption, iron deficiency usually persists in untreated patients due to ongoing hemolysis and subsequent hemoglobinuria.

Although C5 inhibitors alleviate most of the IVH and iron loss, the ongoing transfusion needs that persist could cause transfusional iron overload and iron retention in the hepatocytes and Kupffer cells of the liver [20,21,22]. In addition to residual transfusion dependency, patients treated with C5 inhibitors develop EVH, leading to iron overload and retention due to increased iron absorption from anemia-driven erythropoiesis due to hemolysis, decreased renal iron excretion due to reduced IVH-mediated hemosiderinuria, and EVH-induced iron accumulation in the reticuloendothelial system [20]. In an analysis of 5 patients with PNH who received C5 inhibitors, all patients had high concentrations of ferritin, an iron storage protein and marker of iron overload, after C5 inhibitor treatment, with some manifesting increased hepatic iron on magnetic resonance imaging (MRI) [19]. In a subsequent study of 36 transfusion-independent patients with PNH, those who received eculizumab had significantly higher ferritin concentrations than patients who did not receive a C5 inhibitor and 13 out of 23 eculizumab treated patients had serum ferritin above the upper limit of the reference range [20]. This iron overload in patients without transfusions ruled out transfusions as the cause of the iron overload [20]. Instead, the iron accumulation correlated with ongoing EVH during terminal complement inhibition [20].

Iron regulation in patients receiving pegcetacoplan or other proximal complement inhibitors for PNH has not been thoroughly investigated. We hypothesized that the reduced transfusion needs and control of EVH in patients receiving pegcetacoplan would improve iron regulation. In this post hoc analysis of eculizumab-treated patients who received pegcetacoplan in PEGASUS, we compared baseline characteristics of patients with and without iron overload and investigated the effects of pegcetacoplan treatment on iron regulation.

## 2. Results

### 2.1. Patient Characteristics

Of the 80 patients randomized in PEGASUS, baseline iron overload (i.e., serum transferrin saturation ≥ 50%) was present in 27 patients (33.8%). The mean age at baseline was 55.4 years for patients with baseline iron overload (*n* = 27) and 53.4 years for patients without baseline iron overload (*n* = 53) (Table 1). Baseline hemoglobin concentrations were well below normal in both groups, as expected per the inclusion criteria. Sex was not evenly distributed between the iron overload groups; men comprised 59.3% of patients with iron overload and 26.4% of patients without iron overload. Transfusion need was identified as a predictor of baseline iron overload. Among patients with available transfusion data, patients with iron overload had received a greater number of transfusions in the 12 months before baseline than did patients without iron overload (mean transfusions, 10.8 vs. 6.4; *p* = 0.02); patients with iron overload also had a higher transfusion burden compared with patients in the overall PEGASUS study population. The number of transfusions received decreased with pegcetacoplan in patients with and without iron overload. While the transfusion burden for patients with iron overload decreased substantially during the trial, they continued to receive more transfusions while on pegcetacoplan treatment compared with patients without iron overload (mean transfusions, 5.8 vs. 3.3).

### 2.2. Resolution of Iron Overload with Pegcetacoplan

**Figure 1:**
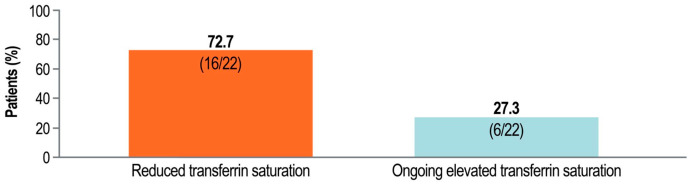
Reduction in transferrin saturation after up to 52 weeks of pegcetacoplan treatment for PNH. PNH, paroxysmal nocturnal hemoglobinuria. Baseline iron overload was defined as a transferrin saturation ≥ 50% at baseline. Resolution of iron overload was defined as a transferrin saturation < 50% after pegcetacoplan treatment. Only patients with evaluable baseline iron overload were included.

Twenty-two of the twenty-seven patients with iron overload at baseline had postbaseline iron overload data. Sustained resolution of iron overload, indicated by reduced transferrin saturation, was achieved within 52 weeks of pegcetacoplan treatment in 16 of these 22 patients (72.7%); iron overload had not resolved in 6 patients (27.3%) (*p* < 0.05) (Figure 1). Of the 16 patients with resolved iron overload, a majority experienced resolution after 20 weeks of pegcetacoplan treatment (*n* = 10); these patients had a mean (SD) transferrin saturation of 34.9% [6.0%]) at week 20. Four patients had iron overload resolution at 32 weeks (mean [SD] transferrin saturation, 32.9% [10.1%]), 1 had resolution at 40 weeks (transferrin saturation, 47.6%), and 1 had resolution at 52 weeks (transferrin saturation, 39.7%).

### 2.3. Iron Markers by Baseline Iron Overload Status

**Figure 2:**
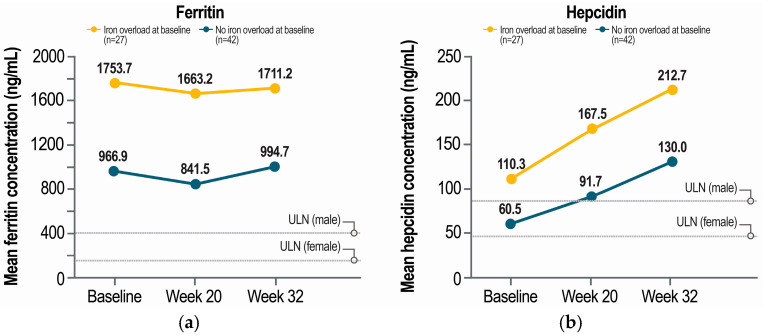
Concentrations of (**a**) ferritin and (**b**) hepcidin in eculizumab-experienced patients with PNH before and after pegcetacoplan treatment, by baseline iron overload status. PNH, paroxysmal nocturnal hemoglobinuria; ULN, upper limit of normal. Baseline iron overload was defined as a transferrin saturation ≥ 50% at baseline. Only patients with available laboratory values at all timepoints were included in the analysis.

**Figure 3:**
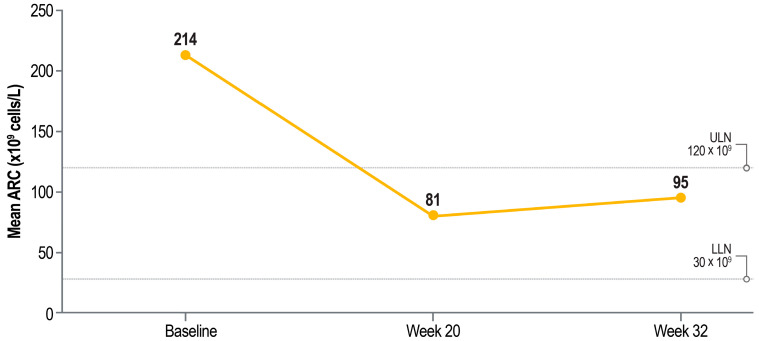
Mean ARCs before and after pegcetacoplan treatment in eculizumab-experienced patients with PNH and baseline iron overload (*n* = 27). ARC, absolute reticulocyte count; LLN, lower limit of normal; PNH, paroxysmal nocturnal hemoglobinuria; ULN, upper limit of normal. Baseline iron overload was defined as a transferrin saturation ≥ 50% at baseline.

Patients with baseline iron overload had ferritin concentrations that were nearly 2 times higher than those of patients without iron overload, and these concentrations were largely maintained in both groups through 32 weeks of pegcetacoplan treatment (Figure 2a). Mean ferritin concentrations were well above normal (upper limits of normal, 400 ng/mL [male patients] and 150 ng/mL [female patients]) throughout the study regardless of baseline iron overload status. At baseline, patients with iron overload had a mean hepcidin concentration of 110.3 ng/mL (above the upper limits of normal of 85.6 ng/mL [male patients] and 47.3 ng/mL [female patients]), and patients without iron overload had a mean hepcidin concentration of 60.5 ng/mL, which is below the upper limit of normal for male patients (Figure 2b). Hepcidin concentrations increased after 20 weeks of pegcetacoplan therapy regardless of iron overload status and further increased at 32 weeks. Mean ARC, an indicator of EVH, decreased from above normal at baseline to within normal limits (30–120 × 10^9^ cells/L) after 20 and 32 weeks of pegcetacoplan treatment (Figure 3).

## 3. Discussion

**Figure 4:**
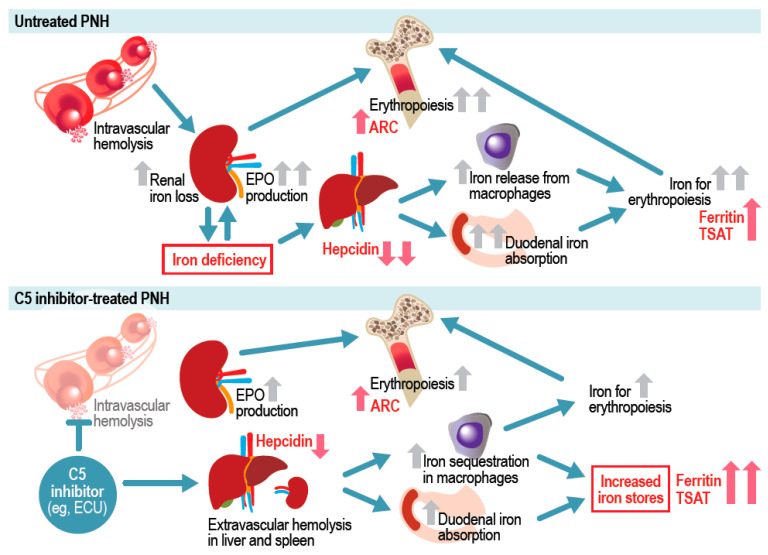
Potential effects of complement C5 inhibitors for PNH on iron regulation. ARC, absolute reticulocyte count; C5, complement factor 5; ECU, eculizumab; EPO, erythropoietin; PNH, paroxysmal nocturnal hemoglobinuria; TSAT, transfusion saturation. Red text indicates iron status markers assessed in the current analysis.

Untreated PNH can result in iron deficiency due to IVH that often requires iron supplementation [19]. However, several previous publications have reported just the opposite (i.e., iron overload) following PNH treatment with the C5 inhibitor eculizumab (Figure 4) [19,20]. In alignment with these findings, the current study identified iron overload at baseline in approximately one-third of patients with PNH who had received eculizumab and experienced residual anemia. The likelihood of iron overload was greater for male patients and for patients with a higher transfusion burden, which aligns with women’s higher propensity for iron deficiency anemia [23] and the recognized potential for iron overload after multiple transfusions [24].

Iron overload with C5 inhibitor use occurs because these terminal complement inhibitors only address the IVH mediated by C5. The proximal portion of the complement cascade is not affected, which means that residual IVH and emergent EVH may still occur [10,11]. As a result, patients may still require chronic blood transfusions, which contribute to further iron accumulation [4]. As noted previously, iron accumulation for patients with PNH with EVH is driven by multiple mechanisms: persistent transfusion dependency, increased iron absorption from anemia-driven erythropoiesis due to hemolysis, decreased renal iron excretion due to reduced IVH-mediated hemosiderinuria, and EVH-induced accumulation in the reticuloendothelial system [20]. It follows that centralized blockade of the complement pathway to prevent EVH, such as that of the C3/C3b inhibitor pegcetacoplan, could improve iron regulation. In the current study, biomarkers of iron overload improved in 72.7% of patients after up to 52 weeks of pegcetacoplan treatment. This resolution often occurred as early as week 20 and was associated with increased mean hepcidin concentrations (a sign predictive of reduced iron absorption). These findings suggest that EVH control and anemia reduction with pegcetacoplan therapy may improve iron regulation in C5 inhibitor–treated patients with PNH. This is further supported by our finding that mean ARC decreased to within the normal range after pegcetacoplan treatment in patients with iron overload.

This study had several limitations. Persistently high ferritin concentrations in eculizumab-experienced patients during 32 weeks of pegcetacoplan may reflect that this period was too short to observe changes in total body iron stores, especially in patients who had been extensively transfused before pegcetacoplan. Additionally, inflammatory and infectious diseases may contribute to elevated ferritin concentrations, which confounds the interpretation of ferritin elevations [25]. Correlative hepatic iron quantitation by MRI was not performed in these patients. In addition, patients were not required to fast before testing, which could have affected their iron levels. Another limitation is that the definition of iron overload was the same for men and women, even though women typically have lower thresholds for anemia than men. Additionally, a lower likelihood of iron overload in females is likely related to the impact of menstruation, and androgens typically lead to higher hemoglobin levels in men but do not affect iron status. Further research into these possibilities is needed.

These limitations reflect the nature of this post hoc analysis of a phase 3 clinical study that was not designed with iron analysis as the priority and could be addressed in a similar study of longer duration with the primary goal of assessing iron regulation. In addition, heritable causes of iron overload were not investigated in this trial. Future research is needed to fully characterize iron dysregulation by assessing additional iron parameters such as MRI quantitation of hepatic iron and markers of inflammation, IVH, and EVH. A comparison of iron regulation in patients who received pegcetacoplan versus a C5 inhibitor could clarify the effects of each treatment and enrich our understanding of the effect of pegcetacoplan on iron dysregulation in patients with PNH.

## 4. Materials and Methods

### 4.1. Patients and Study Design

**Figure 5:**
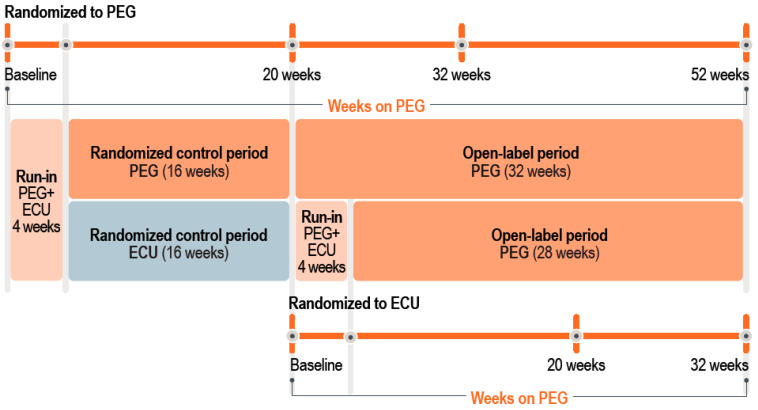
Study design of the PEGASUS trial and the iron parameters analysis. ECU, eculizumab; PEG, pegcetacoplan.

In PEGASUS, a randomized, open-label, phase 3, active comparator, controlled trial, adults with PNH who had residual anemia (hemoglobin concentration < 10.5 g/dL) after ≥3 months of eculizumab treatment received eculizumab and pegcetacoplan for 4 weeks (run-in) and were then randomized (1:1) to eculizumab or pegcetacoplan monotherapy for 16 weeks (to week 20) (Figure 5). This was followed by a 32-week, open-label period, during which patients either continued pegcetacoplan (for a total pegcetacoplan treatment duration of up to 52 weeks) or switched from eculizumab to pegcetacoplan for up to 32 weeks of pegcetacoplan therapy. Blood samples were collected at all study visits, and patients did not fast before blood sampling. Additional trial details have been published elsewhere [14,15].

### 4.2. Post Hoc Analysis

This post hoc study analyzed PEGASUS patient data, including patient records, transfusion-related data, and concentrations of biomarkers such as transferrin, hepcidin, and ferritin assessed throughout the study. Baseline was defined as prior to the run-in for pegcetacoplan-randomized patients and week 20 (i.e., before pegcetacoplan initiation) for eculizumab-randomized patients. Patients with serum transferrin saturation ≥ 50% were defined as having iron overload [26].

### 4.3. Outcomes

Patients’ age, sex, baseline hemoglobin concentration, and transfusion details (i.e., the number of transfusions and units received before baseline and during PEGASUS and the number of red blood cell transfusions in the prior year) were summarized for those with vs. without baseline iron overload to identify factors that differed by iron overload status. In patients with iron overload, the resolution of iron overload (defined as serum transferrin saturation < 50%) was measured after 20, 32, 40, and 52 weeks of pegcetacoplan treatment. Mean biomarker values at baseline and after pegcetacoplan treatment were compared for patients with and without iron overload at baseline. ARC in patients with baseline iron overload was assessed at baseline and after 20 and 32 weeks of pegcetacoplan treatment.

### 4.4. Statistical Analysis

Continuous variables are presented as means and standard deviations or as medians and interquartile ranges. Bivariate comparisons of variables of interest were conducted across various patient-level metrics. Metrics, such as test scores and iron overload status across time on treatment and by treatment groups, were assessed. All statistical analyses were conducted using Excel.

### 4.5. Ethics

The PEGASUS study protocol was designed and monitored in accordance with the ethical principles of Good Clinical Practice and the Declaration of Helsinki [14,15]. These protocols were approved by an institutional review board or independent ethics committee at each center. All patients provided written informed consent before undergoing study-related procedures.

## 5. Conclusions

Our findings identify iron overload in C5 inhibitor–treated patients with PNH. Men and patients with a high transfusion burden may be at a greater risk for iron overload when treated with C5 inhibitors compared with women and those with a low transfusion burden. We show that pegcetacoplan improves biomarkers of iron overload, including reduced transferrin saturation, increased hepcidin concentrations, and decreased ARCs, by blocking both IVH and EVH and preventing anemia. These findings support the importance of being aware of the potential for iron overload during C5 inhibitor treatment and highlight that pegcetacoplan may be used as a treatment option for patients with PNH and iron overload.

## Figures and Tables

**Table 1:** Baseline characteristics of patients with PNH with and without baseline iron overload.

Baseline Characteristics	Patients with Baseline Iron Overload (*n* = 27)	Patients Without Baseline Iron Overload (*n* = 53)	*p*-Value
Age, mean, years	55.4	53.4	NS
Male, *n* (%)	16 (59.3)	14 (26.4)	0.006
Hemoglobin concentration at baseline, ^a^ mean, g/dL	8.6	8.7	NS
Transfusions, mean, *n*			
Before baseline	(*n* = 24) 10.8	(*n* = 33) 6.4	0.02
During the trial	(*n* = 19) 5.8	(*n* = 27) 3.3	NS
Volume transfused, mean, U			
Before baseline	(*n* = 24) 18.1	(*n* = 33) 12.1	NS
During the trial	(*n* = 19) 11.5	(*n* = 27) 6.4	NS
Red blood cell transfusions in the past year, mean, *n*	9.7	6.1	NS

NS, not significant; PNH, paroxysmal nocturnal hemoglobinuria; U, units. ^a^ Normal reference ranges: 12–16 g/dL (female patients) and 13.6–18 g/dL (male patients).

## Data Availability

The datasets generated during and/or analyzed during the current study are available from the corresponding author on reasonable request.

## Abbreviations

The following abbreviations are used in this manuscript:

ARC	Absolute reticulocyte count
C3	Complement factor 3
C5	Complement factor 5
EVH	Extravascular hemolysis
IVH	Intravascular hemolysis
MRI	Magnetic resonance imaging
PNH	Paroxysmal nocturnal hemoglobinuria
